# Impact of Protein Citrullination by Periodontal Pathobionts on Oral and Systemic Health: A Systematic Review of Preclinical and Clinical Studies

**DOI:** 10.3390/jcm13226831

**Published:** 2024-11-13

**Authors:** Marco Bonilla, Natividad Martín-Morales, Rocío Gálvez-Rueda, Enrique Raya-Álvarez, Francisco Mesa

**Affiliations:** 1 Higher Technician in Clinical and Biomedical Laboratory, Centro de Investigación Biomédica (CIBM), 18016 Granada, Spain; 2 Department of Pathology, School of Medicine, University of Granada, 18016 Granada, Spain; nati@ugr.es; 3 Instituto de Investigación Biosanitaria (IBS Institute), 18012 Granada, Spain; 4 Independent Researcher, 18071 Granada, Spain; rgalvez@correo.ugr.es; 5 Department of Medicine, School of Medicine, University of Granada, 18016 Granada, Spain; enriraya@gmail.com; 6 Department of Rheumatology, San Cecilio University Clinical Hospital, 18006 Granada, Spain; 7 Department of Periodontics, School of Dentistry, University of Granada, 18071 Granada, Spain; fmesa@ugr.es

**Keywords:** peptidylarginine deiminase, *Porphyromonas gingivalis*, *Aggregatibacter actinomycetemcomitans*, immunomodulation, citrullination, autoimmune diseases

## Abstract

**Background**: This review synthesizes the role of *Porphyromonas gingivalis* (*P. gingivalis*) and *Aggregatibacter actinomycetemcomitans* (*A. actinomycetemcomitans*) in modulating immune responses through citrullination and assesses its impact on periodontitis and systemic conditions. **Methods:** A systematic review was conducted on preclinical and clinical studies focusing on *P. gingivalis-* and *A. actinomycetemcomitans*-induced citrullination and its effects on immune responses, particularly inflammatory pathways, and systemic diseases. The search included PubMed, Scopus, Google Scholar, Web of Science, and gray literature. Quality and risk of bias were assessed using OHAT Rob Toll and QUIN-Tool. The review is registered in PROSPERO (ID: CRD42024579352). **Results:** 18 articles published up to August 2024 were included. Findings show that *P. gingivalis* and *A. actinomycetemcomitans* citrullination modulates immune responses, leading to neutrophil dysfunction and chronic inflammation. Key mechanisms include citrullination of antimicrobial peptides, CXCL10, histone H3, α-enolase, and C5a, impairing neutrophil activation and promoting NET formation. **Conclusions:** This review suggests that *P. gingivalis* and *A. actinomycetemcomitans* citrullination modulates immune responses and may influence periodontitis and systemic conditions like RA. Beyond ACPA production, these pathogens affect key proteins such as H3, C5a, and CXCL10, as well as antimicrobial peptides, NET formation, and phagocytosis. These interactions lead to neutrophil dysfunction and potentially affect other cells, subsequently disrupting local and systemic inflammatory responses.

## 1. Introduction

In the etiopathogenesis of periodontitis, there are two key causal factors: dysregulation of the host’s inflammatory response and the interaction with a dysbiotic subgingival biofilm [1]. The disease is characterized by a chronic infiltrate mainly composed of lymphocytes, plasma cells, and macrophages, often organized in clusters within the lamina propria surrounding vascular structures [2], typically associated with dysbiosis, a microbial imbalance where pathogenic bacteria outcompete commensal species [3].

Bacteria within the oral microbiome are essential to maintaining microbial homeostasis, yet dysbiosis is strongly associated with periodontal disease progression, which affects approximately 61.6% of individuals globally based on confident case definitions [4]. The commensal microbiota performs essential roles in maintaining oral tissue function and immunity, whereas eubiosis reflects a balanced microbial state resistant to pathogenic shifts. Dysbiosis, however, disrupts the host-microbe equilibrium, facilitating pathogenic microbial behavior and the development of periodontal disease through a community-driven rather than a single-species model [5]. *Pathobionts* (a symbiont that can promote pathology only when specific genetic or environmental conditions in the host are altered) contribute to periodontal tissue destruction through different virulence factors, including proteolytic enzymes and immune evasion mechanisms, which facilitate its long-term survival in host tissues [6,7]. The host’s immune response to persistent dysbiotic plaque biofilms activates inflammatory signaling pathways, leading to tissue breakdown. Key mediators, such as proinflammatory cytokines (e.g., TNF-α, IL-1β), receptor activators of nuclear factor-kappa B ligand and matrix metalloproteinases, contribute to connective tissue and bone degradation, underpinning the clinical presentation of periodontitis [8]. Periodontitis is linked epidemiologically with various comorbidities, including cardiovascular disease, type-2 diabetes, rheumatoid arthritis (RA), inflammatory bowel disease, and Alzheimer’s disease, among others. One possible mechanism behind the association between periodontitis and inflammatory comorbidities is periodontitis-associated low-grade systemic inflammation, a common feature in many chronic conditions. Conversely, systemic diseases can also influence periodontitis, as seen with type-2 diabetes, which may worsen periodontal disease by increasing the inflammatory burden and altering the periodontal microbiome [9].

**Figure 1:**
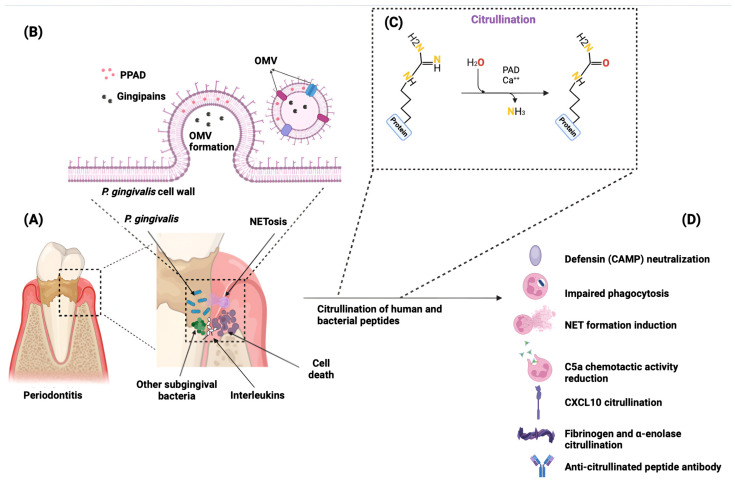
Schematic representation that illustrates the pathway of citrullination by *Porphyromonas gingivalis*. (**A**) The periodontal pocket undergoing an inflammatory process, highlighting the increase in *P. gingivalis* and inflammatory mediators such as interleukins. (**B**) The formation of outer-membrane vesicles (OMVs) containing peptidylarginine deiminase (PPAD) and gingipains, which serve as vehicles for virulence factors. (**C**) Process of citrullination itself. (**D**) Main immune responses triggered by citrullination. Original figure created using BioRender©.

To date, *Porphyromonas gingivalis* (*P. gingivalis*) and *Aggregatibacter actinomycetemcomitans* (*A. actinomycetemcomitans*) remain the only periodontal pathogens conclusively shown to induce citrullination. Investigations into other periodontal bacteria have yet to demonstrate the presence of citrullinating enzymes or the ability to induce citrullination via secreted products. Understanding the interplay between *P. gingivalis* and dysbiosis is crucial for developing targeted therapies [10]. As dysbiosis progresses, it recruits neutrophils, macrophages, interleukins, and other inflammatory mediators to the site of inflammation (Figure 1A). Neutrophils may form neutrophil extracellular traps (NETs)—web-like structures of chromatin fibers and antimicrobial proteins (e.g., histones, neutrophil elastase, and myeloperoxidase)—that capture and neutralize harmful bacteria [11]. Although NETs are crucial for controlling bacterial infections, their release can also inflict collateral damage to periodontal tissues, potentially exacerbating tissue destruction [12].

Evidence shows that *P. gingivalis* strains expressing active gingipains robustly induce NETs formation [13]. Recent findings have highlighted that peptidylarginine deiminase 4 (PAD4) is critical for NET formation induced by *P. gingivalis*, as it facilitates histone citrullination, which is necessary for chromatin decondensation and NET release [14]. Additionally, *P. gingivalis* can modify host proteins through citrullination, also known as deimination, a post-translational modification that influences immune responses and contributes to the progression of periodontitis. During citrullination, the PAD enzyme converts the amino acid arginine into citrulline, a process dependent on high calcium levels first described by Rogers and Simmonds in 1958 [15]. Citrullination involves the hydrolysis of the positive charge of arginine, producing neutral citrulline and urea (Figure 1C). This alteration in charge can affect protein structure, disrupt protein-protein interactions, and lead to protein denaturation. Citrullination can occur across various cellular proteins, including those in the membrane, cytoplasm, nucleus, and mitochondria [16].

*P. gingivalis* expresses a unique enzyme, *Porphyromonas* peptidylarginine deiminase (PPAD), which is responsible for this citrullination process. This enzyme is localized in the outer membrane of *P. gingivalis* and exists in the extracellular environment in a soluble form. It is also associated with outer membrane vesicles (OMVs) [17] (Figure 1B). A prominent consequence of peptide citrullination mediated by *P. gingivalis* is the development of anti-citrullinated peptide antibodies (ACPAs). Following infection with *P. gingivalis*, as discussed earlier, neutrophils undergo NET formation and are then enriched with active proteases and PADs, which facilitate the generation of citrullinated epitopes, subsequently driving the production of ACPAs, closely associated with RA [18] and can appear in serum years before the first outbreak of RA, with their presence being associated with more severe RA and a worse prognosis [19]. In contrast, other consequences of peptide citrullination by *P. gingivalis* remain less well-documented in the literature. Given the emerging evidence linking peptide citrullination to various pathophysiological processes, a systematic review of this topic is crucial.

Furthermore, *A. actinomycetemcomitans* is another pathogen closely linked to the pathogenesis of periodontal disease, particularly with aggressive periodontitis [20]. Like *P. gingivalis*, *A. actinomycetemcomitans* has been implicated in systemic conditions, including RA. One of the key virulence factors of *A. actinomycetemcomitans* is Leukotoxin A (LtxA), a potent toxin that selectively targets host immune cells, particularly neutrophils and macrophages, leading to their lysis. By impairing these substantial components of the host immune defense, LtxA facilitates an imbalance in the microbial community, ultimately driving dysbiosis in the subgingival environment [21].

The objective of this systematic review is to collect, evaluate, and summarize all available information on the mechanisms by which *P. gingivalis* and *A. actinomycetemcomitans* induce citrullination and describe their potential impact on human physiological processes, including immune modulation and systemic inflammatory responses.

## 2. Materials and Methods

### 2.1. Protocol and Registration

A systematic review protocol was prepared during the planning stages and registered with PROSPERO (ID: CRD42024579352). The review was designed and conducted in accordance with the PRISMA checklist (Preferred Reporting Items for Systematic Reviews and Meta-Analyses) [22]. The PRISMA guidelines are attached as a Supplementary Document.

### 2.2. Eligibility Criteria

For this review, two PICO strategies were utilized. The first PICO strategy addressing *P. gingivalis* was employed to guide this study and answer the research question: “How does peptide citrullination by *P. gingivalis* compare to non-citrullinating strains in terms of their biological effects on different levels, including the potential to induce systemic inflammatory responses or autoimmune diseases”? The PICO strategy was: P (experimentation with *P. gingivalis* that includes citrullination process), I (peptide-citrullination induced by *P. gingivalis*), C (non-citrullinating bacteria or no comparison group), O (effects of citrullinated peptides at different levels of the organism, including potential systemic effects).

The second PICO strategy focused on investigating the role of *A. actinomycetemcomitans* in citrullination and periodontitis pathogenesis. The research question was: “What are the biological effects of *A. actinomycetemcomitans* and its Leukotoxin A on the host’s immune response and periodontal health, including the potential for systemic inflammatory responses?” The PICO strategy for this question was: P (experimentation with *A. actinomycetemcomitans*), I (effect of LtxA produced by *A. actinomycetemcomitans*), C (non-citrullinating bacteria or no comparison group), O (effects of LtxA on the host’s immune response and peptide citrullination).

Clinical and preclinical studies that evaluated the effect of peptide citrullination by *P. gingivalis* and *A. actinomycetemcomitans* were included. The literature search imposed no restrictions on the year of publication, language, or publication status. The following studies were not included: (i) reviews, letters to the editor, book chapters, abstracts, case reports, and editorials; and (ii) studies with insufficient or irrelevant data on the citrullination pathway under investigation.

### 2.3. Information and Search Sources

The following databases were utilized for information gathering PubMed (including MedLine), Scopus, Google Scholar, and Web of Science. To capture the ‘gray literature’ and minimize selection and publication bias, additional sources such as OpenGrey, OpenThesis, LILACS, and TESEO were also consulted. The search was conducted independently by two reviewers (M.B. and R.G.R.) from 1 July to 30 August 2024. Medical Subject Headings (MeSH) were employed to select relevant keywords. The search strategy was enhanced using Boolean operators ‘AND’ and ‘OR’, resulting in the following search terms: (citrullination OR deimination OR PPAD OR peptidyl arginine deiminase) AND (periodontopathogens OR periodontal bacteria OR *Porphyromonas gingivalis* OR *Aggregatibacter actinomycetemcomitans*) AND (systemic pathology OR systemic disease OR autoimmune diseases).

### 2.4. Selection of Studies

The study selection process was conducted in two phases by two independent reviewers (M.B. and R.G.R.). In the first phase, titles and abstracts of records were reviewed to determine their relevance to the research topic. In the second phase, full-text articles of the selected records were read to ensure they met the inclusion criteria. Studies not adhering to these criteria were excluded. Inter-examiner reliability was assessed using Kappa’s Agreement Coefficient, which yielded a value of 0.921, indicating a high level of agreement between the reviewers. In cases of disagreement, a third reviewer (F.M.) was consulted to make the final decision and resolve inconsistencies. This approach ensured the selection process was rigorous and unbiased.

### 2.5. Data Extraction

The two reviewers independently assessed the selected articles based on the preferred reporting items for systematic review and meta-analyses (PRISMA) [23]. Relevant data were extracted from the studies, including objective, bacteria to be analyzed, and inclusion of a control group were documented. If these two authors encountered any disagreement, a third reviewer (F.M.) was called upon to resolve the discrepancy.

### 2.6. Risk of Bias and Quality Assessment

The risk of bias was evaluated using two methodologies: (i) the OHAT Rob toll [24] (Table 1), (ii) QUIN Tool. QUIN Tool was also utilized to assess the quality of the studies, specifically designed for studies in Dentistry [25] (Table 2). Quality and risk assessments were performed by the two authors. Any discrepancies were resolved through a discussion between M.B. and R.G.R.

## 3. Results

### 3.1. Risk of Bias and Quality Assessment

The OHAT Rob Toll analyses indicated that most articles exhibited a risk of bias related to blinding and outcome assessment (Table 1). In the QUIN-Tool analysis, 11 articles (61%) were classified as having a medium risk of bias (score 50–70%), 5 articles (28%) were classified as having a low risk of bias (score > 70%), and 2 articles (11%) were classified as having a high risk of bias (score < 50%).

### 3.2. Study Selection

**Figure 2:**
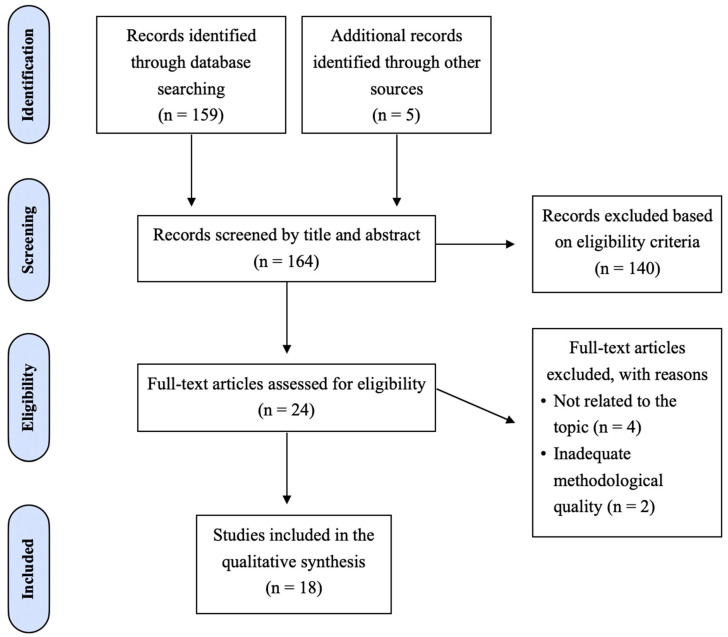
Flowchart of study selection for the systematic review.

The flowchart of the study selection process is reported in Figure 2. The electronic search resulted in the identification of 159 studies and 5 additional articles from Gray Literature. Notably, in this initial stage, articles reporting both *P. gingivalis* and *A. actinomycetemcomitans* were searched. During the first phase, 140 articles were excluded after reading their titles and abstracts, leaving 24 articles to be fully analyzed. After full-text reading, 6 more articles were excluded, 2 of them for inadequate methodological quality and 4 studies for not being entirely related to the theme. A total of 18 studies were included in the review.

### 3.3. Study Characteristics

Table 3 provides an overview of the general characteristics of the included studies, detailing the bacterial strains and control groups employed in each, while Figure 1D highlights the most relevant findings related to human physiology as reported in the studies concerning *P. gingivalis*.

One of the key factors contributing to the pathogenicity of *P. gingivalis* is its role in modulating the biofilm’s structure and bacterial movement [27,31]. In the absence of *ppad* gene (Δppad), there is an increase in biofilm formation due to an increase in matrix production and gingipain-derived adhesin proteins. The enhanced biofilm formation observed in Δppad strains underscores the enzyme’s role in facilitating surface translocation by reducing adhesin proteins in the matrix.

Notably, PPAD contained within OMVs was found to interfere with the immune response [32,39]. The enzyme was found to citrullinate cationic antimicrobial peptides (CAMPs), C5a, and histone H3, thereby impairing NETs formation and reducing the chemotactic activity of C5a and its capacity to activate neutrophils. The citrullination activity observed in OMVs of *P. gingivalis* is dependent on functional PPAD, with no citrullinated proteins detected in the *Δppad* strain [28].

Moreover, three articles [32,36,37,43] highlighted the interaction between PPAD and gingipains (RgpA and Kgp) as crucial to the full virulence of *P. gingivalis*. These studies demonstrated that gingipains not only generate substrates for PPAD by cleaving proteins at arginine residues, such as fibrinogen and α-enolase but are also citrullinated by PPAD, subsequently impairing phagocytosis. Maresz et al. demonstrated that mice infected with *P. gingivalis* showed earlier onset and more severe arthritis, as well as higher MPO activity, indicating more neutrophil infiltration. Higher levels of antibodies against citrullinated α-enolase were found in the serum of mice infected with *P. gingivalis* [42]. Reichert et al. found anti-citrullinated α-enolase antibodies slightly elevated in patients with periodontitis [38]. Importantly, Abdullah et al. demonstrated that PPAD activity remains unaffected by gingipain inhibition, indicating that PPAD and gingipains function independently in terms of their enzymatic activities [41].

Elkaim et al. demonstrated that *P. gingivalis* enhances its virulence by suppressing human PADs while upregulating its own PPAD enzyme, leading to the formation of new citrullinated peptide bands (15–45 kDa) in infected cells, thereby disrupting immune homeostasis [34]. In line with this, Hamamoto et al. showed that *P. gingivalis*, particularly via oral infection, exacerbates arthritis by increasing IL-6 levels and citrullinated peptides in systemic tissues. In fecal-microbiota-transplantation-transferred mice, *P. gingivalis* colonization resulted in severe joint destruction, higher arthritis scores, and systemic inflammation, underscoring the pathogen’s dual local and systemic inflammatory potential [30]. Furthermore, strains exhibiting elevated PPAD activity were more frequently found in patients with moderate to advanced periodontitis [26].

In addition, *P. gingivalis* was found to citrullinate immune-related chemokines [29,40]. Aliko et al. demonstrated that *P. gingivalis* led to an upregulation of genes involved in IL-1β, CXCL8, CCL20, and IL36G. However, Moelants et al. found that while CXCL10 was rapidly degraded by *P. gingivalis*, IL-8 exhibited minimal citrullination, reinforcing PPAD’s selective targeting.

**Figure 3:**
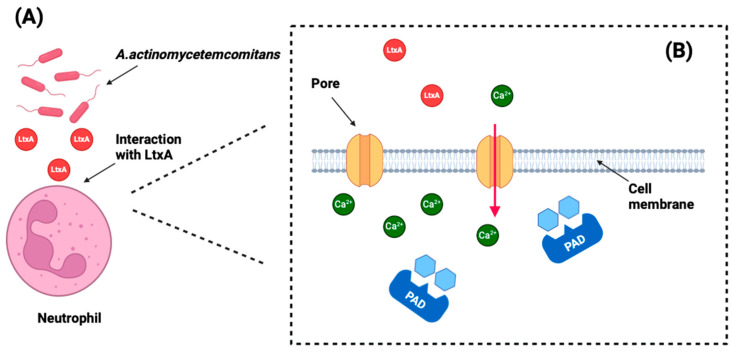
Schematic representation of the citrullination pathway induced by *Aggregatibacter actinomycetemcomitans* (*A. actinomycetemcomitans*). (**A**) *A. actinomycetemcomitans* secretes leukotoxin A (LtxA), which targets and interacts with neutrophils. (**B**) LtxA forms pores in the neutrophil membrane, disrupting intracellular calcium homeostasis. This abnormal calcium influx hyperstimulates the host’s peptidylarginine deiminase (PAD) enzymes, leading to increased citrullination of proteins. Original figure created using BioRender©.

Engström et al. found that citrullinated proteins by *A. actinomycetemcomitans* were predominantly present in gingival tissues of periodontitis patients (80%) compared to healthy controls (27%), with no significant differences in citrullination between the epithelial compartments of periodontitis and healthy tissues. PAD2 and PAD4 levels were also higher in periodontitis, indicating a strong link with *A. actinomycetemcomitans* infection [33]. Konig et al. further demonstrated that LtxA from *A. actinomycetemcomitans* induces hypercitrullination in neutrophils by creating pores in the cell membrane, which allows uncontrolled calcium influx into the cells [35] (Figure 3).

## 4. Discussion

### 4.1. Impact on Systemic Health

To our knowledge, no systematic review has comprehensively synthesized the mechanistic insights into *P. gingivalis*- and *A. actinomycetemcomitans*-mediated citrullination and its role in immune modulation. Although significant research has been conducted on *P. gingivalis* as a keystone pathogen in periodontitis, classified in the red complex by Socransky [44,45], and its association with RA, there remains a notable gap in reviews addressing the mechanisms of PPAD-mediated citrullination and its impact on immunomodulation. Similarly, *A. actinomycetemcomitans* has been linked to several systemic conditions, such as infectious endocarditis, brain abscesses, and chest wall abscesses [46]. Although earlier studies proposed *A. actinomycetemcomitans* as the principal pathogen in localized aggressive periodontitis, recent insights indicate that it plays a crucial role as part of a microbial consortium associated with the disease [47]. This review addresses this gap by integrating the current knowledge on how citrullination by *P. gingivalis* and *A. actinomycetemcomitans* modulate immune responses and their role in the evolution of diseases such as periodontitis and RA.

Recent studies have uncovered additional immunological and systemic effects of citrullination. The *ppad* gene has been found to impair biofilm formation and enhance bacterial motility. This dual effect facilitates the progression of surface translocation [27], aiding in the colonization of epithelial surfaces within the buccal mucosa [48].

Furthermore, *P. gingivalis* has the capacity to create OMVs and release them into the environment. OMVs are linked to the bacterial stress response, such as during the colonization of the host tissues [49]. Zhou et al., in 1998, proposed a model explaining Gram-negative bacteria OMVs formation. During bacterial growth, the cell wall is excised and separated from the peptidoglycan, releasing muramyl peptides. These peptides generate turgor pressure on the outer membrane. If this pressure is not alleviated by the reabsorption of the muramyl peptides, it leads to the continuous formation of expanding blebs, which ultimately detach and are shed into the growth medium [50]. As described by Bielecka et al. and Stobernack et al. 2018, OMVs contain PPAD and gingipains, thereby serving as a vehicle for virulence factors and inducing immune responses [32,39,51]. As previously mentioned, OMV-transported PPAD can citrullinate complement factors, such as C5a [39], due to the presence of C-terminal citrulline, hence reducing its chemotactic activity and neutrophil activation. In severe inflammatory responses, like periodontitis, large numbers of neutrophils are released. However, when neutrophil activation is affected, the ability of neutrophils to respond to infections effectively is reduced, leading to a weakened immune response and potentially contributing to the progression of chronic inflammatory conditions [52]. Stobernack et al. 2018 found that PPAD helps *P. gingivalis* evade neutrophil evasion by citrullinating gingipains, thus contributing to phagocyte dysfunction [32]. Defects in phagocytosis lead to the accumulation of unphagocytosed remnants that disrupt tissue homeostasis as it exerts cytotoxicity on cells [53].

Stobernack et al. 2018 also found that PPAD citrullinates histone H3 (citH3). CitH3 has been demonstrated to be more toxic to endothelial cells than H3 and to induce prominent intracellular leakage [54]. Recent studies have found that citH3 triggers NETosis [55] (Figure 1), which, as previously mentioned [12], can be harmful to periodontal tissues, as an exaggerated NET formation releases reactive superoxide species and proteolytic enzymes [56]. The pathway through which *P. gingivalis* reaches the nucleus to citrullinate H3 remains under investigation. Given that PPAD, with a molecular weight of 47-85 kDa, is a relatively small protein, we hypothesize that PPAD can enter host cells via OMVs through clathrin-dependent, caveolin-dependent, or lipid raft-mediated endocytosis [57] and subsequently reach the nucleus, where it exerts its citrullination activity on H3.

Antimicrobial peptides (AMPs) facilitate the elimination of pathogenic microorganisms, fungi and viruses [58]. Inside this broad group of peptides, CAMPs represent a specific type of AMPs characterized by their net positive charge. CAMPs attract negatively charged bacterial membranes, leading to the elimination of bacteria by disrupting their membranes [59]. The antimicrobial effect of CAMPS depends on their cationic nature; therefore, when CAMPs are citrullinated, their antibacterial and LPS-neutralizing capacities are abrogated [60].

CXCL10 is an inflammatory cytokine that binds to CXCR3 and activates and recruits T cells, eosinophils, monocytes, and NK cells [61]. When CXCL10 is citrullinated, it becomes less effective in activating signaling pathways in cells expressing the CXCR3 receptor. Specifically, citrullinated CXCL10 fails to induce the phosphorylation of ERK1/2 and AKT, which are key kinases in cellular signaling [62]. CXCL8 binds to CXCR2 and induces chemotaxis in neutrophils and other granulocytes [63,64]. However, when CXCL8 is citrullinated, its signaling potency and chemotactic activity are reduced, potentially leading to weaker neutrophil activation [65].

In addition, Stobernack et al. 2016 and Bereta et al. found that *P. gingivalis* in severe periodontitis and RA exerts a more virulent effect by worsening periodontal values (clinical attachment loss and probing pocket depth) and citrullinating specific proteins, thereby exerting a more virulent role. [26,36]

*P. gingivalis* is unique in its ability to citrullinate peptides using its own enzyme, PPAD. In contrast, *A. actinomycetemcomitans* follows a different pathogenic strategy (Figure 3). This bacterium triggers hypercitrullination by secreting LtxA, a virulence factor that induces hypercitrullination by disrupting the neutrophil membrane, leading to calcium influx, PAD4 activation, and the release of citrullinated proteins and virulence factors, thereby contributing to autoimmunity in the pathogenesis of RA [21,33,35].

Following these major findings, we hypothesize that citrullination by *P. gingivalis* and *A. actinomycetemcomitans* plays a pivotal role in immunomodulation by shaping an immune landscape that contributes to disease progression and immune dysfunction. This alteration leads to a weakened immune environment and reduced effectiveness in immune responses, which promote a rapid progression of infections and potentially exacerbate conditions like RA. By targeting key immune regulators, such as CAMPs, cytokines, and C5a, *P. gingivalis* facilitates neutrophil dysfunction in various ways, reducing neutrophil activation and phagocytosis. Furthermore, the citrullination of C5a diminishes its chemotactic activity, thereby compromising the immune response to infections. Collectively, these mechanisms highlight the complex interplay between *P. gingivalis* and the host immune system, emphasizing how the bacterium’s virulence strategies extend beyond local periodontal tissue damage to influence systemic inflammatory and autoimmune responses. Similarly, *A. actinomycetemcomitans* contributes to this immune dysfunction through the secretion of LtxA. This process not only affects local periodontal tissue but also influences systemic inflammatory and autoimmune responses. Collectively, these mechanisms highlight the complex interplay between *P. gingivalis*, *A. actinomycetemcomitans*, and the host immune system, emphasizing how these bacteria’s virulence strategies extend beyond local damage to impact systemic diseases. This review underscores the need for further research to unravel these interactions and explore potential therapeutic strategies targeting citrullination pathways to mitigate the impact of these pathogens in periodontal and systemic diseases.

### 4.2. Impact on Oral Health

In periodontitis, *P. gingivalis* utilizes OMVs to deliver virulence factors like PPAD and gingipains. PPAD citrullinates complement factors such as C5a, impairing their ability to recruit and activate neutrophils due to alterations in chemotaxis and phagocytosis, which weakens the immune response and exacerbates chronic inflammation [32,39,55]. Additionally, PPAD citrullinates H3, which is toxic to endothelial cells and induces NETosis, releasing reactive superoxide species and proteolytic enzymes that further damage periodontal tissues [12,54].

In addition to *P. gingivalis*, *A. actinomycetemcomitans* induces hypercitrullination through the secretion of LtxA. This virulence factor disrupts neutrophil membranes, leading to the activation of PAD4 and increased citrullination of proteins. Citrullination by PAD4 is also essential for chromatin decondensation during the formation of NETs, which exacerbate myocardial damage during ischemia-reperfusion injury [66]. This process disrupts the endothelial barrier, increasing vascular permeability, and promotes thrombus formation by facilitating interactions with molecules such as the Von Willebrand factor [67]. Consequently, CitH3 contributes to microvascular obstruction, delayed healing, and prolonged inflammation, all of which are essential factors in worsening cardiac function [68]. Given that *P. gingivalis* can citrullinate H3 via its PPAD enzyme and *A. actinomycetemcomitans* can hyperactivate PAD4 through LtxA, we hypothesize that these pathobionts may interact in the context of cardiovascular diseases through mechanisms similar to those described above; however, further research in this area is necessary to elucidate these potential interactions. Notably, citrullination of specific proteins, such as H3 and C5a, may serve as new prognostic indicators, particularly as the citrullination capacity of *P. gingivalis* is heightened in advanced periodontitis. Therefore, targeting *P. gingivalis* and *A. actinomycetemcomitans* could offer therapeutic opportunities to address neutrophil dysfunction and mitigate systemic effects associated with periodontal diseases.

However, this review has limitations. Some studies had small sample sizes, which may impact the generalizability of the findings, and there was a lack of control groups in certain cases. Additionally, a meta-analysis could not be conducted due to the nature of the reviewed studies, which, rather than presenting conflicting results, each emphasized different systemic effects of PPAD-mediated citrullination. This review enriches the scientific database by synthesizing knowledge on *P. gingivalis* and *A. actinomycetemcomitans* in periodontal disease, highlighting their mechanisms and clinical implications. It identifies research gaps, guiding future studies, and improving clinical strategies while fostering interdisciplinary dialogue. Although this review provides valuable insights, further in vivo research is needed to confirm these findings and explore their broader clinical implications. Notably, some clinical studies reviewed did not specify the proteins affected by citrullination.

## 5. Conclusions and Future Perspectives

This review suggests that *P. gingivalis* and *A. actinomycetemcomitans* citrullination modulates immune responses and may influence periodontitis and systemic conditions like RA. Beyond ACPA production, these pathogens affect key proteins such as H3, C5a, and CXCL10, as well as antimicrobial peptides, NET formation, and phagocytosis. These interactions lead to neutrophil dysfunction and potentially affect other cells, subsequently disrupting local and systemic inflammatory responses. By identifying research gaps, this review aims to guide future studies and refine clinical strategies, while fostering interdisciplinary dialogue to advance understanding and treatment approaches.

## Figures and Tables

**Table 1:** Risk of bias from OHAT Rob Toll.

Question Author	Was Administered Dose or Exposure Level Adequately Randomized?	Was Allocation to Study Groups Adequately Concealed?	Were Experimental Conditions Identical Across Study Groups?	Were Research Personnel Blinded to the Study Group During the Study?	Were Outcome Data Complete Without Attrition or Exclusion from the Analysis?	Can We Be Confident in the Exposure Characterization?	Can We Be Confident in the Outcome Assessment (Including Blinding of Assessors)?	Were There No Other Potential Threats to Internal Validity?
Bereta GP et al. (2024) [26]	−	++	++	−	+	+	−	+
Vermilyea DM et al. (2021) [27]	−	++	++	−	++	+	−	+
Larsen DN et al. (2020) [28]	−	++	++	−	+	+	−	+
Aliko A et al. (2020) [29]	−	++	++	−	++	+	−	+
Hamamoto Y et al. (2020) [30]	−	++	+	−	++	+	−	+
Vermilyea DM et al. (2019) [31]	−	++	++	−	++	+	−	+
Stobernack T et al. (2018) [32]	−	++	++	−	++	+	−	+
Engström M et al. (2018) [33]	−	++	++	−	++	+	−	+
Elkaim R et al. (2017) [34]	−	++	++	−	++	+	−	+
Konig MF et al. (2016) [35]	−	++	++	−	+	+	−	+
Stobernack et al. (2016) [36]	−	++	++	−	+	+	−	+
Montgomery AB et al. (2016) [37]	−	++	++	−	+	+	−	+
Reichert S et al. (2015) [38]	−	++	++	−	++	+	−	+
Bielecka E et al. (2014) [39]	−	++	++	−	++	+	−	+
Moelants EAV et al. (2014) [40]	−	++	+	−	+	+	−	+
Abdullah SN et al. (2013) [41]	−	++	++	−	++	+	−	+
Maresz K et al. (2013) [42]	−	++	++	++	+	+	−	−
Wegner N et al. (2011) [43]	−	++	+	−	++	+	−	+

(++) there is direct evidence to affirm the answer to the question; (+) there is indirect evidence to affirm the answer to the question; (−) there is indirect evidence to respond negatively to the question.

**Table 2:** Quality Assessment of the Studies by QUIN-Tool.

Quality Assessment Tool for In Vitro Studies													
	Clearly Stated Aims	Detailed Explanation of Sample Size Calculation	Detailed Explanation of Sampling Technique	Details of Comparison Group	Detailed Explanation of Methodology	Operator Details	Randomization	Method of Measurement Outcome	Outcome Assessor Details	Blinding	Statistical Analysis	Presentation of Results	Score (%)
Bereta GP et al. (2024) [26]	++	−	+	++	++	−	−	++	+	−	+	+	50
Vermilyea DM et al. (2021) [27]	+	NA	++	++	++	−	−	++	+	NA	+	++	65
Larsen DN et al. (2020) [28]	++	NA	++	++	++	−	−	++	+	NA	NA	++	83
Aliko A et al. (2020) [29]	++	NA	++	++	++	−	−	++	+	NA	++	++	75
Hamamoto Y et al. (2020) [30]	+	++	++	++	++	−	−	++	+	−	++	++	75
Vermilyea DM et al. (2019) [31]	−	NA	++	++	++	−	−	++	+	NA	NA	++	61
Stobernack T et al. (2018) [32]	++	NA	++	++	++	−	−	++	+	NA	++	++	75
Engström M et al. (2018) [33]	++	−	+	++	++	−	−	++	+	++	++	++	67
Elkaim R et al. (2017) [34]	++	NA	++	++	++	−	−	++	+	NA	+	++	70
Konig MF et al. (2016) [35]	++	−	−	++	−	−	−	++	+	−	+	++	42
Stobernack et al. (2016) [36]	++	NA	++	++	++	−	−	++	+	NA	++	++	75
Montgomery AB et al. (2016) [37]	++	NA	++	++	++	−	−	++	+	NA	NA	++	72
Reichert S et al. (2015) [38]	++	−	+	++	++	−	−	++	+	−	++	++	58
Bielecka E et al. (2014) [39]	+	NA	++	++	++	−	−	++	+	NA	++	++	70
Moelants EAV et al. (2014) [40]	−	−	+	NA	++	−	−	++	+	−	+	+	40
Abdullah SN et al. (2013) [41]	++	NA	++	++	++	−	−	++	+	NA	NA	+	67
Maresz K et al. (2013) [42]	−	−	+	++	++	−	−	+	+	+	++	++	50
Wegner N et al. (2011) [43]	++	−	++	NA	++	−	−	++	+	−	NA	++	55

(++) Adequately specified; (+) Inadequately specified; (−) Not specified; NA (Not applicable); >70% = low risk of bias; 50% to 70% = medium risk of bias; <50% = high risk of bias.

**Table 3:** Summary of the general characteristics of the included articles.

Author/Year	Strain	Bacteria Provenance	Control Group (Non Citrullinating)	Citrullination Ability	Main Techniques Employed	Periodontal Effects	Systemic Effects
**Clinical Studies on *P. gingivalis***							
Bereta GP et al. (2024) [26]	Not specified	Clinical isolates	*P. gingivalis* W83 and ATCC 22377	Yes	PCR, Western Blot, HPLC, RNA-seq	Advanced chronic periodontitis showed more citrullinating activity. CAL and PPD values correlate with the presence of *P. gingivalis* strain harboring a triple polymorphic variant (G231N, E232T, N235D) *P. gingivalis* strains with higher PPAD activity were found more frequently in subjects with moderate/advanced and advanced CP	Not applicable
Stobernack et al. (2016) [36]	*P. gingivalis* ATCC 33277 and W83 and three isolates: 20658, MDS16, MDS45	American Type Culture Collection and clinical isolates (CP-RA)	*P. Gingivalis* W83 and TCC 33277Δppad	Yes	Mass spectrometry, exoproteome analysis	The RA-associated isolates MDS16 and MDS45 showed unique citrullinated proteins, such as the Mfa1 fimbrilin. Gingipains and other key virulence factors like RgpA were citrullinated in specific isolates like W83 and MDS45, but not in PPAD-deficient mutants or other strains	Not applicable
Reichert S et al. (2015) [38]	Not specified	Clinical isolates	Non-RA non-Periodontitis patients	Yes	ELISA, HLA typing, PCR	Anti- α-enolase antibodies were slightly elevated in the periodontitis group.	Not applicable
Moelants EAV et al. (2014) [40]	*P. gingivalis* ATCC 33277, 5 clinical isolates *(P. gingivalis* to *P. gingivalis5)*	Research laboratory and clinical isolates	Protease inhibitors, Five capsule-typed strains (K1 to K5)	Yes	Enzymatic assay, ELISA	Not applicable	No citrullination was detected in CCL3, which lacks NH2-terminal Arg and hence could not be sequenced for citrullination. CXCL8 incubated with different strains of *P. gingivalis* showed minimal citrullination (up to 5%). CXCL10 levels rapidly decreased, indicating rapid degradation by *P. gingivalis*
Wegner N et al. (2011) [43]	*P. gingivalis* W83, 4 clinical isolates (MaRL, D243, JH16, J430)	Research laboratory and clinical isolates (CP)	*P. gingivalis* mutants (Δ*ppad*, *ppad*+, Δ*rgp*, Δ*kgp*, Δ*rgp*+*kgp*), *Prevotella intermedia* H13 (clinical isolate), *Prevotella oralis* ATCC 33269, *Capnocytophaga gingivalis* ATCC 33624, *Capnocytophaga ochracea* ATCC 27872	Yes	Immunoblot, HPLC, mass spectrometry	*P. gingivalis* expresses endogenous citrullinated proteins, a feature absent in other oral bacteria tested. The presence of citrullinated proteins in *P. gingivalis* is entirely dependent on the PPAD.	Arginine-gingipains play a crucial role in generating substrates for PPAD by cleaving proteins at arginine residues. *P. gingivalis* can citrullinate human proteins such as fibrinogen and α-enolase after cleavage by arginine-gingipains
**Clinical Studies on *A. actinomycetemcomitans***							
Engström M et al. (2018) [33]	Not specified	Clinical isolates from periodontal patients	Healthy controls	Yes	Immunohistochemistry, qPCR	Citrullinated proteins were found predominantly in gingival tissues of periodontitis patients (80%) versus healthy controls (27%). No significant differences in citrullination were noted between the epithelial compartments of periodontitis and healthy tissues. PAD2 and PAD4 expressed higher levels in periodontitis.	Not applicable
Konig MF et al. (2016) [35]	Not specified	Clinical isolates from RA patients	Healthy controls	Yes	PCR, ELISA, mass spectrometry	Not applicable	LtxA induces hypercitrullination in neutrohpils by creating pores in the cell membrane, allowing uncontrolled calcium influx into the cell.
**In Vivo Studies on Laboratory Animals on *P. gingivalis***							
Hamamoto Y et al. (2020) [30]	*P. gingivalis* W83 and 33277	American Type Culture Collection	PPAD knockout	Yes	ELISA, pirosequencing		FMT from *P. gingivalis*-inoculated mice exacerbated joint destruction and increased IL-6 and CP levels in recipient mice. *P. gingivalis* oral infection led to severe joint destruction, elevated arthritis scores (AS), and increased production of IL-6 and citrullinated peptides (CP) in serum, joint, and intestinal tissues.
Maresz K et al. (2013) [42]	*P. gingivalis* W83	Not specified	PPAD knockout, *Prevotella Intermedia* and control mice not inoculated with *P. gingivalis*	Yes	PCR, immunoscan, MPO determination	Not applicable	Mice infected with live *P. gingivalis* showed earlier onset and more severe arthritis compared to control mice. Higher MPO activity, indicating more neutrophil infiltration, was observed in joints of *P. gingivalis*-infected mice. Higher levels of antibodies against citrullinated α-enolase were found in the serum of mice infected with live *P. gingivalis*.
**In Vitro Studies on *P. gingivalis***							
Vermilyea DM et al. (2021) [27]	*P. gingivalis* 381	H. Kuramitsu, State University of Buffalo, Buffalo, NY and American Type Culture Collection	*P. gingivalis* 381 Δ*ppad*	Yes	Enzymatic and biofilm assay, quantification of OMVs, RNA-seq, PCR	PPAD plays a crucial role in regulating biofilm dynamics, specifically by facilitating surface translocation and reducing biofilm formation. *PPAD plays a key role in regulating sessile lifestyle towards surface translocation.*	*P. gingivalis* 381 Δ*ppad accumulates more intracellular arginine (lacks PPAD activity) and creates fewer and smaller OMVs.*
Larsen DN et al. (2020) [28]	*P. gingivalis* W83	Not specified	*P. gingivalis* W83 Δ*ppad and* C351A (cysteine mutated to alanine)	Yes	HPLC separation, mass spectrometry, aminoacid assay	*P. gingivalis* W83 exhibited more citrullination compared to the mutant strain.	Citrullination in OMVs is dependent on functional PPAD, no citrullinated proteins were identified in the ΔPPAD strain and only one in the PPADC351A mutant.
Aliko A et al. (2020) [29]	*P. gingivalis* WT 33277	American Type Culture Collection	*P. gingivalis* 33277 Δ*ppad and* PPADC351A (inactive)	Yes	PCR, flow cytometry, SEM, RNA-Seq	PPAD activity does not affect the ability of *P. gingivalis* to adhere to or be internalized by human oral keratinocytes.	The gene expression analysis revealed that PPAD activity in *P. gingivalis* specifically affects immune response pathways in oral keratynocites, notably impacting IL-1 signaling and immune cell chemotaxis. Key genes such as CXCL8, IL36G, CCL20, and IL1B showed significant PPAD-dependent expression changes, underscoring PPAD’s role in modulating immune responses.
Vermilyea DM et al. (2019) [31]	*P. gingivalis* 381	State University of Buffalo, Buffalo, NY	*P. gingivalis* Δ8820	Yes	TEM, cryo-SEM, enzymatic assay, immunoblot	Δ*ppad* in *P. gingivalis* enhances biofilm formation. PPAD inhibits biofilm formation by regulating matrix production. This enhanced biofilm formation is not due to increased fimbriae expression but is linked to the absence of citrullination in key proteins like RgpA and Kgp. Δ8820 biofilms contained more gingipain-derived adhesin proteins and more matrix.	PPAD impacts growth, colonization, attachment, and invasion of host cells and tissues.
Stobernack T et al. (2018) [32]	*P. gingivalis* W83	American Type Culture Collection and clinical isolates (CP-RA)	*P. gingivalis* W83 Δppad	Yes	Immunohistochemistry, phagocytosis assay, flow cytometry, mass spectrometry	Not applicable	The presence of PPAD significantly impairs the binding and internalization of P. gingivalis by neutrophils. PPAD interferes with the phagocytosis process by citrullinating gingipains, which are involved in modulating actin polymerization and phagocytosis. PPAD citrulliantes histone H3, impairing NETosis, it also citrullinates LP9 and therefore neutralizes CAMPs.
Elkaim R et al. (2017) [34]	*P, gingivalis* 33277	American Type Culture Collection	Uninfected human chondrocytes	Yes	qPCR, immunoblot, enzymatic assay	Not applicable	*P. gingivalis* can suppress the expression and activity of human PADs while enhancing the activity of its own PAD enzyme. Infection with live *P. gingivalis* resulted in new citrullinated peptide bands (15 kDa to 45 kDa) in cellular extracts.
Montgomery AB et al. (2016) [37]	*P. gingivalis* W83	Not specified	*P. gingivalis* tPPADC351A, tPPADR152A, tPPADR154A,	Yes	Enzymatic assay, mass spectormetry	Not applicable	The Cys351Ala mutation rendered the enzyme catalytically inactive, Cys351 plays a key role in PPAD’s enzymatic function. PPAD specifically citrullinates C-terminal arginine residues, distinguishing its substrate specificity from human PAD2 and PAD4, which prefer internal and N-terminal arginine residues. tPPADWT citrullinated peptides derived from RA autoantigens, such as fibrinogen and α-enolase.
Bielecka E et al. (2014) [39]	*P. gingivalis* W83	Not specified	*P. gingivalis* W83 Δppad	Yes	Isolation of OMV, HPLC, mass spectrometry, calcium mobilization assay	Not applicable	PPAD citrullinates C5a. OMVs from *P. gingivalis* W83 efficiently citrullinate C5a, thus indicating that OMVs carry both Arg-specific gingipains and PPAD. C5a-Cit showed significantly reduced chemotactic activity for neutrophils. At high concentrations and/or prolonged incubation with PPAD, C5a capacity to activate neutrophils is abrogated.
Abdullah SN et al. (2013) [41]	*P. gingivalis* W50	Research laboratory	Protease inhibitors	Yes	Gingipains inhibition, azocasein assay, colorimetric assay, protein assay	Optimal activity was observed between pH 7.5 and 8, with significant activity retained at pH 9. PPAD exhibited 87% of its activity in heat-treated cells, indicating heat stability. Vimentin showed the highest rate of citrullination.	Gingipain inhibition did not affect PPAD activity. PPAD and gingipains function independently in terms of their enzymatic activities.

*P. gingivalis, Porphyromonas gingivalis; A. actinomycetemcomitans, Aggregatibacter actinomycetemcomitans*; CP, Chronic periodontitis; *CAL*, Clinical Attachment Loss; *PPD*, probing pocket depth; *Δppad*, deletion of the ppad gene; *PPAD*, peptidylarginine deiminase; *OMV*, outer-membrane vesicle; *RA*, Rheumatoid Arthritis; *tPPAD*, truncated-PPAD; FMT, Fecal microbiota transplantation.

## Supplementary Materials

The following supporting information can be downloaded at: https://www.mdpi.com/article/10.3390/jcm13226831/s1, PRISMA guidelines, list of abbreviations.
