# Supplementary material for: Impact of Protein Citrullination by Periodontal Pathobionts on Oral and Systemic Health: A Systematic Review of Preclinical and Clinical Studies
Source: J Clin Med. 2024 Nov 13;13(22):6831. doi: 10.3390/jcm13226831 (PMC11594594; doi:10.3390/jcm13226831)
Supplement: Supplementary file 1 [file jcm-13-06831-s001.zip › abbreviation list.pdf]

ACPA: Anti-citrullinated peptide antibody – an antibody produced in response to citrullinated proteins, often associated with rheumatoid arthritis.

AMP: Antimicrobial peptide – small proteins that contribute to the innate immune response by destroying microbial membranes.

C5a: Complement component 5a – an anaphylatoxin that plays a crucial role in the inflammatory response by recruiting immune cells to sites of infection or injury.

CAMP: Cationic antimicrobial peptide – a positively charged antimicrobial peptide that interacts with negatively charged microbial membranes to eliminate pathogens.

cit-: A prefix denoting the presence of citrulline or the process of citrullination, often used in the context of proteins modified by peptidylarginine deiminases.

CXCL8: C-X-C motif chemokine 8 – a chemokine involved in the recruitment of immune cells during inflammation, particularly neutrophils.

CXCL10: C-X-C motif chemokine 10 – a chemokine involved in the recruitment of immune cells during inflammation and infection.

H3: Histone H3 – a protein component of chromatin that plays a role in the structural organization of DNA and can be modified to regulate gene expression.

LtxA: Leukotoxin A – a toxin produced by *A. actinomycetemcomitans* that targets immune cells, contributing to periodontal disease.

NETs: Neutrophil extracellular traps – web-like structures formed by neutrophils containing chromatin and antimicrobial proteins, capturing and neutralizing pathogens.

OMV: Outer membrane vesicle – small, membrane-bound vesicles released by bacteria, often containing virulence factors.

PAD: Peptidylarginine deiminase – an enzyme that catalyzes the citrullination process by converting arginine to citrulline.

PPAD: Porphyromonas peptidylarginine deiminase – a unique PAD enzyme expressed by *P. gingivalis*, involved in protein citrullination and linked to immune modulation.

RA: Rheumatoid arthritis – a chronic autoimmune disorder that causes inflammation and joint damage, often linked to periodontal pathogens.

A. actinomycetemcomitans: *Aggregatibacter actinomycetemcomitans* – a bacterial species associated with aggressive periodontitis, known for producing leukotoxin A.

P. gingivalis: *Porphyromonas gingivalis* – a keystone pathogen in periodontitis known for its role in dysbiosis and production of virulence factors, such as PPAD.
